# The Roles of a Multidrug-Resistant *Klebsiella pneumoniae* High-Risk Clone and Its Resistance Plasmids on the Gastrointestinal Colonization and Host-Defense Effectors in the Gut

**DOI:** 10.3390/antibiotics13080698

**Published:** 2024-07-26

**Authors:** Balazs Stercz, Judit Domokos, Zsuzsanna A. Dunai, Nora Makra, Janos Juhasz, Eszter Ostorhazi, Bela Kocsis, Dora Szabo

**Affiliations:** 1 Institute of Medical Microbiology, Semmelweis University, 1089 Budapest, Hungary; stercz.balazs@semmelweis.hu (B.S.); domokos.judit@semmelweis.hu (J.D.); makra.nora@semmelweis.hu (N.M.); juhaszjanos4@gmail.com (J.J.); ostorhazi.eszter@semmelweis.hu (E.O.); kocsis.bela@semmelweis.hu (B.K.); 2 HUN-REN-SU Human Microbiota Research Group, 1052 Budapest, Hungary; zsuzsanna.dunai@gmail.com; 3 Faculty of Information Technology and Bionics, Pázmány Péter Catholic University, 1083 Budapest, Hungary; 4 Neurosurgical and Neurointervention Clinic, Semmelweis University, 1083 Budapest, Hungary

**Keywords:** colonization, gut, multidrug resistance, mouse model, defensins, microbiome, ESBL, CTX-M, OXA-carbapenemase

## Abstract

The asymptomatic gastrointestinal colonization of multidrug-resistant (MDR) bacteria can lead to difficult-to-treat infections. We investigated the role of host factors influencing colonization in an orogastrical murine infection model using a CTX-M-15- and OXA-162-producing *Klebsiella pneumoniae* ST15 (MDR-KP) strain, as well as *Escherichia coli* J53 (EC) and *E. coli* transconjugants with an IncFII(K) plasmid carrying CTX-M-15 (EC-CTXM), and with an IncL plasmid carrying OXA-162 (EC-OXA) genes. The fecal bacterial count in colony-forming unit/gram stool (CFU/g) was determined by cultivation, IgA and defensin levels by ELISA, and gut microbiota by 16S rRNA analysis. The CFU was the lowest in EC, followed by EC-OXA and EC-CTXM, and the highest in the MDR-KP group. The IgA level in feces increased in MDR-KP, EC-CTXM, and EC-OXA, and did not change in EC. The beta-defensin 3 level markedly increased in all groups, with the highest values in MDR-KP and EC-CTXM. Alpha-defensin-5 increased in all groups especially in EC. In microbiota, the *Bacteroidota* phylum was dominant in MDR-KP, EC-CTXM, and EC-OXA, whereas *Proteobacteria* was dominant in EC. The *Muribaculaceae* family was significantly more common in the MDR-KP and EC-OXA groups, while the *Lachnospiraceae* family was dominant in the EC group. While fecal IgA levels positively correlated with colonizing bacterial CFU, the alpha-defensin 5 levels inversely correlated with CFUs and IgA levels. The presence of the IncFII(K) plasmid induced beta-defensin 3 production. The amounts of the *Muribaculaceae* family members exhibited a correlation with the IncL plasmid. The detected amounts of the *Lachnospiraceae* family indicated the protective role against the high-risk clone and the resistance plasmids’ dissemination. Our results suggest that not only the MDR-KP clone itself but also the resistance plasmids play a primary role in the colonization rate in the gastrointestinal tract. Both the MDR-KP clone as well as the IncFII(K) and IncL resistance plasmids provide survival and colonization benefits in the gut.

## 1. Introduction

The global spread and increasing prevalence of multidrug-resistant (MDR) *Enterobacterales* pose a significant threat to our healthcare systems. These Gram-negative pathogens often resist commonly used third-generation cephalosporins and carbapenems due to the production of extended-spectrum β-lactamases (ESBLs), such as CTX-M types, and carbapenemases, including KPC, metallo-beta-lactamase, and OXA-type carbapanemase [1]. Making antibiotic-resistance dissemination more widespread, the genes encoding these ESBLs and carbapenemases are located on mobile genetic elements [2]. Infections caused by ESBL- or carbapenemase-producing *Enterobacterales* are associated with increased morbidity and mortality rates compared to infections caused by less resistant organisms [3]. In response to this growing threat, the World Health Organization in 2018 designated both ESBL-producing and/or carbapenemase-producing *Enterobacterales* as critical priority pathogens for the research and development of new therapeutic strategies and rapid diagnostics [4].

Since *Enterobacterales* are common commensal bacteria of the intestinal microbiota, infections caused by MDR-*Enterobacterales*—particularly, *Escherichia coli* and *Klebsiella pneumoniae*—often originate from prior asymptomatic gut colonization. The relationship between the intestinal microbiota and IgA production, as well as defensin production, has been investigated in connection with several primarily gastrointestinal diseases [5,6,7,8]. Defensins help to maintain the balance of microbiota by controlling the growth of pathogens and promoting the survival of beneficial bacteria [9]. Generally, IgA production in the gut plays a crucial role in the immune response. *K. pneumoniae*, as a common gut pathobiont, can induce intestinal inflammation [10]. However, the pathogenicity of *Klebsiella* is sensitive to the colonization status of gut microbiota [5].

High-risk *K. pneumoniae* clones are detected worldwide in hospital settings, and these are capable of acquiring diverse antibiotic-resistance mechanisms that enable them to survive in the hospital environment. Furthermore, high-risk clones can asymptomatically colonize the gut, and these clones are responsible for a high number of difficult-to-treat infections, because these exhibit multidrug resistance; therefore, limited number of effective antibiotics are available for treatment [11,12].

*K. pneumoniae* ST15 is an internationally disseminated high-risk clone that has been identified globally and appears to be resistant to multiple antibiotics, including cephalosporins, carbapenems, and fluoroquinolones. The high prevalence and virulence of *K. pneumoniae* ST15 strains make them a significant clinical and public health concern, particularly in hospital settings where they can spread rapidly and induce outbreaks [13,14,15].

CTX-M-15-producing *K. pneumoniae* ST15 is a widely disseminated clone that has been identified globally, particularly in Europe. The clone has been found to be highly transferable and has undergone multiclonal spread, contributing to its widespread presence in different parts of Europe. In the context of hospital settings, the presence of IncFII(K) plasmids carrying CTX-M-15 can contribute to the spread of multidrug-resistant bacteria, strongly limiting treatment options. IncL plasmids are often associated with antimicrobial-resistance genes, such as OXA-type carbapenemase [2,16].

The purpose of the current study was to determine different host factors influencing the gastrointestinal colonization of multidrug-resistant *Enterobacterales* strains. Our goal was to examine separately the role of resistance plasmids during colonization. We aimed to assess the effects of intestinal colonization with a CTX-M-15 ESBL- and OXA-162 carbapenemase-producing *K. pneumoniae* ST15 high-risk clone; and a sensitive, laboratory *E. coli* J53 strain and its transconjugants—either with a CTX-M-15-harboring IncFII(K) plasmid or with an OXA-162-harboring IncL plasmid—in a murine model to quantify the effects of the *K. pneumoniae* ST15 high-risk clone itself and the resistance plasmids on the establishment and elimination of intestinal colonization [17].

**Figure 1:**
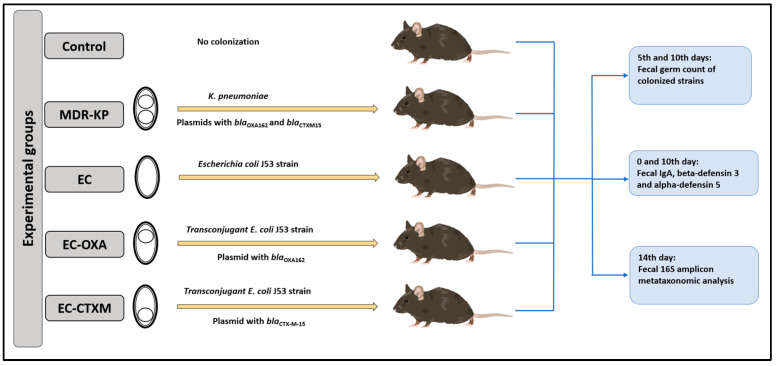
Experimental design.

One particular aim of our study was to perform gastrointestinal colonization in oral-ampicillin-pretreated mice (C57BL/6) with a clinical CTX-M-15 ESBL- and OXA-162 carbapenemase-producing *K. pneumoniae* ST15 strain (MDR-KP), as well as its *E. coli* J53 transconjugants with an IncFII(K) plasmid containing the *bla*_CTX-M-15_ resistance gene (EC-CTXM), *E. coli* J53 transconjugants with an IncL plasmid containing the *bla*_OXA-162_ resistance gene within a Tn1991.2 genetic element (EC-OXA), and *E. coli* J53 strain itself (EC) [17]. After the orogastric colonization of the mice with the strains listed above, the bacterial count of the feces for the colonizing bacteria, the content of IgA, and the levels of beta-defensin-3 and alpha-defensin-5 were determined at different time points (Figure 1).

## 2. Results

### 2.1. Bacterial Loads in Feces During Gastrointestinal Colonization

**Figure 2:**
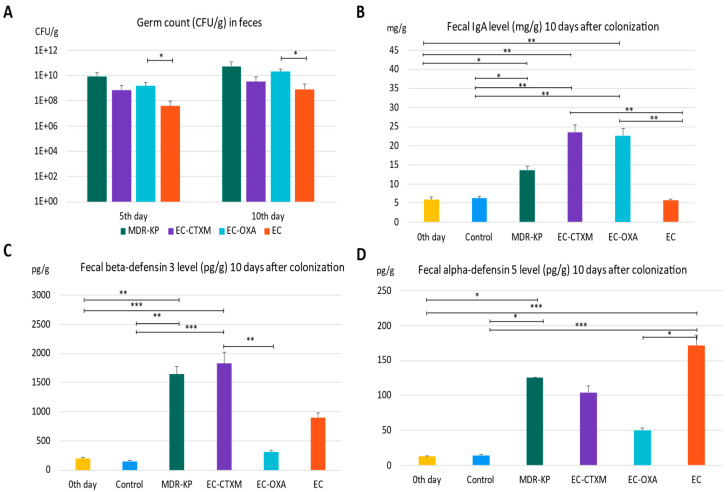
(**A**) The gastrointestinal colonization rate characterized by germ count (colony-forming units: CFU) in the feces by different bacteria—MDR-KP, EC, EC-OXA, and EC-CTX-M—on the fifth and tenth day of colonization. (**B**) The IgA level in feces of mice colonized by different bacteria MDR-KP, EC, EC-OXA, and EC-CTXM. (**C**) The beta-defensin-3 level in feces of mice colonized by different bacteria MDR-KP, EC, EC-OXA, and EC-CTXM. (**D**) The alfa-defensin-5 in feces of mice colonized by different bacteria MDR-KP, EC, EC-OXA, and EC-CTXM. Statistical differences are marked with * *p* < 0.05; ** *p* < 0.01; and *** *p* < 0.001.

The mice were colonized orogastrically with MDR-KP (CTX-M-15- and OXA-162-producing *K. pneumoniae* ST15 strain), EC (*E. coli* J53), EC-CTXM (*E. coli* J53 transconjugant with an IncFII(K) plasmid containing the *bla*_CTX-M-15_ resistance gene), and EC-OXA (*E. coli* J53 transconjugant with an IncL plasmid containing the *bla*_OXA-162_ resistance gene within a Tn1991.2 genetic element). Fecal samples were collected on the fifth and tenth day of the colonization in order to determine the colonizing bacteria amount in the feces. On the fifth day of colonization, the CFU was the lowest at 3.77 × 10^7^ CFU/g in the EC group, indicating the low colonization capability of laboratory-sensitive strains. The colonization rate was the highest in the MDR-KP group with a mean value of 8.64 × 10^9^ CFU/g, and was also high in the EC-CTXM group at 7.06 × 10^8^ CFU/g and in the EC-OXA group at 1.54 × 10^9^ CFU/g, indicating the elevated gastrointestinal colonization ability of the transconjugant *E. coli* strains. The determination of germ counts from stool samples taken on the tenth day after colonization exhibited similar trends. On the tenth day, the fecal mean bacterial load was in the MDR-KP group at 5.43 × 10^10^ CFU/g, in the EC group at 8.43 × 10^8^ CFU/g, in the EC-CTXM group at 3.18 × 10^9^ CFU/g, and in the EC-OXA group at 2.01 × 10^10^ CFU/g (Figure 2A).

### 2.2. Fecal IgA Levels During Gastrointestinal Colonization

The IgA level was measured in feces just before the colonization (mean of 5.89 mg/g) and it was increased in the MDR-KP group (mean of 13.67 mg/g) and did not change in the EC group (mean of 5.66 mg/g). However, it markedly increased in the group EC-CTXM (mean of 22.68 mg/g) and in the group EC-OXA (mean of 23.52 mg/g) by Day 10 (Figure 2B).

### 2.3. Fecal Beta-Defensin 3 Levels During Gastrointestinal Colonization

In the feces, the baseline mean beta-defensin 3 level was a mean of 196.63 pg/g. The beta-defensin-3 level was increased to a mean of 1640 pg/g in the MDR-KP group, to a mean of 898 pg/g in the EC group, to a mean of 309 pg/g in the EC-OXA group, and to a mean of 1825 pg/g in the EC-CTXM group, indicating the dominant effect of the *bla*_CTX-M-15_-containing IncFII(K) plasmid on beta-defensin 3 production (Figure 2C).

### 2.4. Fecal Alpha-Defensin Levels During Gastrointestinal Colonization

The level of alpha-defensin 5 increased to a mean of 125 pg/g in the MDR-KP group, to a mean of 172 pg/g in the EC group, to a mean of 104 pg/g in the EC-CTXM group, and to a mean of 50 pg/g in the EC-OXA group, and it was increased in the EC groups. Based on these results, the fecal alpha-defensin 5 levels were inversely correlated with CFUs and IgA levels (Figure 2D).

### 2.5. Fecal Microbiota Composition During Gastrointestinal Colonization

**Figure 3:**
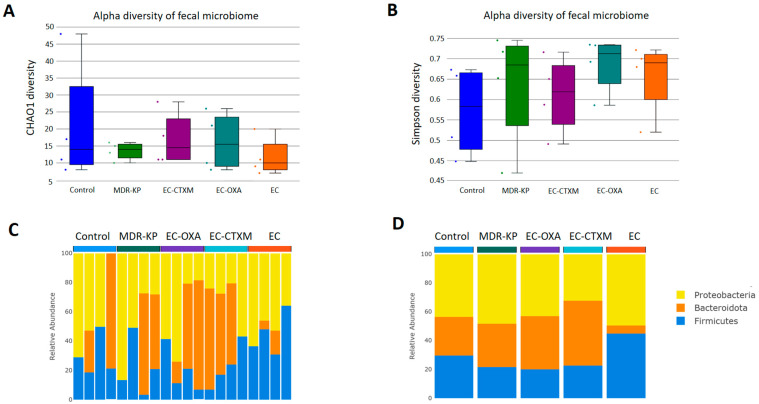
(**A**) Relative abundances of abundant taxonomic phylum in each mouse. Elements are shown if they have at least 2% relative abundance in at least one of the averaged samples. (**B**) Average values of relative abundances at the phylum level were calculated for samples from the same treatment groups. Elements are shown if they have at least 2% relative abundance in at least one of the averaged samples. (**C**) Chao1 alpha-diversity of fecal samples in the different groups (Control, MDR-KP, EC, EC-CTXM, and EC-OXA). Box plots show the distribution of diversities in each group. (**D**) Simpson alpha-diversity of fecal samples in the different groups (Control, MDR-KP, EC, EC-CTXM, and EC-OXA). Box plots show the distribution of diversities in each group.

**Figure 4:**
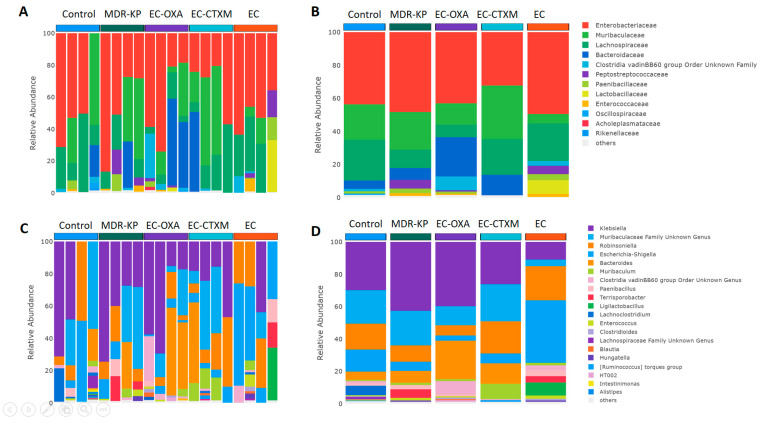
(**A**) Relative abundances of most abundant taxonomic families in each mouse. Elements are shown if they have at least 2% relative abundance. (**B**) Average values of relative abundances at the family level were calculated for samples from the same treatment groups. Elements are shown if they have at least 2% relative abundance in at least one of the averaged samples. (**C**) Relative abundances of most abundant taxonomic genera in each mouse. Elements are shown if they have at least 2% relative abundance. (**D**) Average values of relative abundances at the genus level were calculated for samples from the same treatment groups. Elements are shown if they have at least 2% relative abundance in at least one of the averaged samples.

A 16S rRNA taxonomic analysis was performed on the feces samples on Day 14. There were no significant differences in the alpha-diversity by the Chao1 and Simpson tests and in the beta-diversity among the groups. The *Bacteroidota* phylum was the most dominant phyla in the MDR-KP, EC-CTXM, and EC-OXA-162 groups, whereas, in the EC group, the *Proteobacteria* phylum was dominant (Figure 3). At the family level, the *Muribaculaceae* family was significantly (*p* < 0.05) more common in the EC-OXA-162 groups than in the EC group, showing a correlation with the presence of the OXA-162 plasmid. The *Lachnospiraceae* family was dominant in the EC group, indicating the protective effect of the *Lachnospiraceae* family against the high-risk *Klebsiella* clone and the CTX-M15- and OXA-162-containing resistance plasmid dissemination (Figure 4).

**Figure 5:**
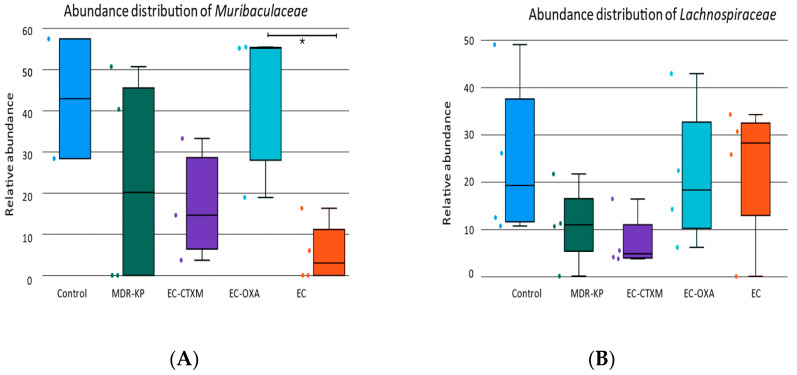
(**A**) The relative abundance of *Muribaculaceae* family in each group (Control, MDR-KP, EC, EC-CTXM, and EC-OXA). (**B**) The relative abundance of *Lachnospiraceae* family in each group (Control, MDR-KP, EC, EC-CTXM, and EC-OXA). Statistical difference is marked with * *p* < 0.05.

On one hand, the abundance of the *Lachnospiraceae* family showed an inverse relationship with the gastrointestinal carriage of the MDR-KP strain and harboring of the resistance plasmid in the EC-CTXM and EC-OXA-162 groups. On the other, the *Enterobacteriaceae* family showed a correlational relationship with the high-risk *Klebsiella* clone and the CTX-M15- and OXA-162-containing resistance plasmids (Figure 5).

## 3. Discussion

Gastrointestinal colonization by multidrug-resistant strains of Enterobacterales has been the focus of attention worldwide because these multidrug-resistant strains (e.g., ESBL- and/or carbapenemase-producing *K. pneumoniae*, and *E. coli*) can spread easily from person to person between healthy individuals as well as among hospitalized patients. The gut environment provides optimal conditions (e.g., a high bacterial density, optimal temperature, and a source of nutrients for bacteria) for horizontal gene transfer, that enhances the further dissemination of resistance genes among intestinal bacteria, and multidrug-resistant strains can evolve [18,19].

Several studies have investigated the risk factors of colonization with multidrug-resistant ESBL- and carbapenemase-producing Enterobacterales, such as earlier antibiotic treatment, previous hospitalization, intensive care unit treatment, travel abroad, etc. [20,21,22,23,24,25,26,27,28,29,30,31]. The screening for intestinal colonization with ESBL- and carbapenemase-producing Enterobacterales strains has been implemented in healthcare settings in several countries. Its importance is well-described among patients (e.g., newborns, and patients who are transferred between hospitals); however, healthy people after travel can be also screened [20,21,22,23,24,25].

Different decolonization strategies have been investigated in order to diminish the intestinal colonization of ESBL- and carbapenemase-producing Enterobacterales strains, because the prior gastrointestinal colonization can induce systemic infections and can initiate community-acquired infections. Furthermore, intestinally carried MDR-Enterobacterales strains can induce several outbreaks in hospitals as well [32].

In our animal study, after the colonization assay, the amount of MDR-*Klebsiella* and the *E. coli* strains harboring the CTX-M or the OXA-162 plasmids markedly increased in feces. Interestingly, the *E. coli* strain without the plasmid was not able to colonize the gastrointestinal tract; however, *K. pneumoniae* and *E. coli* harboring different resistance plasmids were able to successfully colonize.

It seems that the presence of the IncFII(K) plasmid with *bla*_CTX-M-15_ and the IncL plasmid with *bla*_OXA-162_ in *E. coli* changed the colonization properties of the original all-sensitive *E. coli* strain. Several underlying mechanisms can explain the reason, that the carriage of plasmids was accompanied by an increase in the bacterial cell count in stool samples.

In recent years, the relationship between the intestinal microbiome composition and ESBL-producing *E. coli* has been investigated. Davies et al. conducted a point-prevalence metagenomics study on fecal samples from international travelers before and after travel, observed changes in the microbiome composition during travel, and found that these changes were primarily associated with the development of travelers’ diarrhea rather than the acquisition of ESBL-producing *E. coli* [33]. In another study, no differences were found in the diversity parameters or relative abundance of bacterial species in the gut microbiome between healthy individuals, who were colonized or not colonized with ESBL-producing *E. coli* [34]. Recently, Ducarmon et al. analyzed the potential role of the gut microbiome in controlling the colonization of ESBL-producing *E. coli*, and no differences in the diversity parameters or in the relative abundance were observed between ESBL-producing *E. coli* and the negative groups [35]. Our obtained results are in good correlation with the previous literature data that describe human results, and we also found no difference in the alpha-diversity between the control group that does not carry ESBL or carbapenemase genes, and the group of ESBL- and carbapenemase-carrying strains [36,37,38]. Based on our findings, the abundance of *Bacteroidota* phylum was correlated with multidrug-resistance features. It was clearly dominant in the colonization with *Klebsiella* or with *E. coli* containing either the ESBL or OXA-162 plasmids. The gut microbiota exhibits remarkable alteration after colonization with the carpanemase-producing *K. pneumoniae* in animal studies with a specific dysbiosis characterized by a consistently marked decrease in *Muribaculaceae*, *Rikenellaceae*, and Lachnospiraceae_NK4A136_group [36]. However, we could not detect a significant difference in the abundance of the *Lachnospiraceae* and *Muribaculaceae* groups. Having said that, a significant difference could be observed in favor of ES and EC-OXA-162 in terms of *Muribaculaceae*, and a small difference could be detected in the EC group in terms of *Lachnospiraceae* in our study. The presence of the OXA-162 plasmids either in the *K. pneuomoniae* or *E. coli* strain led to changes in the gut microbiota composition, with a *Muribaculaceae* dominance, showing a correlation with the presence of the plasmid. The *Muribaculaceae* family specializes in the fermentation of complex polysaccharides. A genomic analysis has also suggested that the capacity for propionate production is widespread in the family [39,40].

Apart from the composition of the microbiome behind the gastrointestinal colonization with multidrug-resistant *Klebsiella* or *E. coli*, we are unaware of other mechanisms investigated in human or animal studies. The role of IgA and defensins in the gastrointestinal tract of mice colonized with a multidrug-resistant clone and its long-term colonization remains to be studied in detail. In order to identify the mechanisms involved in the gastrointestinal colonization of multidrug-resistant strains, we quantified the IgA and beta-defensin levels in mouse feces.

IgA binds to commensal bacteria and pathobionts like *Klebsiella*, which, in turn, can inhibit their growth and penetration of the mucus layer [40,41]. Interestingly, *Klebsiella* itself can induce IgA production in the gut [5]. Persistent colonization by resistant *E. coli* induces the secretion of luminal IgA, while commensal *E. coli* strain does not [40,41]. However, our results demonstrate that host immunity selectively recognizes pathobiont *E. coli* with the specific resistance plasmids, and develop specific IgA. The induced IgA specific to resistant *E. coli*, in turn, contributes to preventing the resistant strains from accessing the epithelium. *K. pneumoniae* induces a targeted IgA response in the gut, which helps to control its own colonization levels [10]. However, *Klebsiella* pathogenicity depends on the overall composition of the gut microbiota, with a dysbiotic state favoring *Klebsiella* overgrowth and inflammation [10,36]. Our results indicate that the presence of resistance plasmids—the IncFII(K) plasmid with CTXM-15 and the IncL plasmid with OXA-162—play a primary role in the MDR colonization rate in the gastrointestinal tract.

*K. pneumoniae* colonization in the gut can induce the production of human beta-defensins, which are antimicrobial peptides that help to regulate the gut microbiome [42,43]. Specifically, *Klebsiella* infection leads to increased levels of human beta-defensin 2 and human beta-defensin 3 in the intestine [44]. However, the total number of specific intestinal microbiota like *Klebsiella* does not differ significantly based on the different beta-defensin levels, suggesting that, while *Klebsiella* induces beta-defensin production, the defensin levels alone do not determine the abundance of *Klebsiella* in the gut [43,44]. Experimentally altering the gut microbiome composition can lead to changes in the beta-defensin-3 secretion, indicating a complex interplay between the microbiome, *Klebsiella* colonization, and host antimicrobial peptide production in the intestine [45]. Our results indicate that *bla*_CTX-M-15_ plays a dominant effect in containing the IncFII(K) plasmid in beta-defensin 3 production.

*E. coli* is known to play a significant role in the production and regulation of defensins in the gut. *E. coli* can induce the production of defensins in response to various stimuli, including the presence of pathogens. Different *E. coli* strains can have distinct effects on defensin production and gut health. For example, the probiotic *E. coli* Nissle 1917 can induce human beta-defensin 2 production, while other strains may have different effects on the gut microbiota [46]. Based on our results, the *bla*_CTX-M-15_-containing IncFII(K) plasmid presence plays an important role in human beta-defensin 3 production. Surprisingly, the alpha-defensin 5 level was the highest in the case of colonization with the apathogen *E. coli* and lower during the colonization with the plasmid carrying strains in this study. These findings highlight the importance of *E. coli* in the production and regulation of defensins in the gut, a process that plays a crucial role in maintaining gut health and controlling the growth of pathogens.

To our knowledge, this is the first paper that studied not only the gut microbiome dynamics, but also the role of IgA production and defensin levels during colonization by a multidrug-resistant *K. pneumoniae* high-risk clone. We documented that IgA levels and human beta-defensin 3 production have a crucial role in colonization and plasmid dissemination. All these findings confirm and emphasize that plasmids carrying resistance genes play a significant role in the spread of high-risk clones worldwide, whose role goes beyond the spread of resistance. The further identification of plasmid-mediated factors involved in colonization requires additional studies.

The limitations of the study are as follows: Further studies have to identify the direct roles of defensins on the pathogenic-resistance plasmid or indirect effects through the microbiota modification. Other MDR high-risk clones of *K. pneumoniae* and *E. coli* that carry different resistant plasmids and resistance genes should be tested in a colonization model.

## 4. Materials and Methods

### 4.1. Bacterial Strain and Conjugation Assay

For colonizing the experimental groups of mice, different bacterial strains were used that were generated previously and described in detail [17]. We provide here a summary of the relevant information related to these experiments. A multiresistant *K. pneumoniae* isolate ST15 (5825) (MDR-KP) was used as a conjugation donor. It harbors different resistance plasmids, among them an IncF(II)K-replicon-type plasmid with the gene *bla*_CTX-M-15_ and an IncL-replicon-type plasmid with the gene *bla*_OXA-162_. MDR-KP were also resistant to ciprofloxacin, that was used as selection agent during cultivation for germ count determination. *E. coli* J53 (EC) strain was used as an acceptor in the conjugation assay. It is resistant to sodium-azide, used as selection agent for acceptor and transconjugant strains. Two transconjugant strain were isolated, one isolate harboring IncL plasmid with *bla*_OXA162_ (EC-OXA) and one isolate harboring IncF(II)K plasmid with *bla*_CTX-M-15_ (EC-CTXM). These four different bacterial strains were propagated on Luria–Bertani (LB) agar (Biolab, Budapest, Hungary) (EC) and LB agar with 8 mg/L ampicillin (Sandoz, Schaftenau, Austria) (MDR-KP, EC-OXA, and EC-CTXM). A suspension in phosphate buffer saline (PBS, VWR, Debrecen, Hungary) was made containing 10^9^ CFU/mL for gastrointestinal colonization of mice.

### 4.2. Animal Study

For gastrointestinal colonization, C57BL/6 male mice (Jackson Laboratory, Bar Harbor, ME, USA), aged 6–8 weeks, were used. The mice were individually housed in standard ventilated cages (IVC) under a 12 h light–dark cycle, with controlled temperature (20–22 °C). They had ad libitum access to sterile food and water, along with sterile bedding material. To prevent potential interference from natural gut bacteria, each mouse was housed individually throughout the experiment. Prior to the commencement of the experiments, the mice underwent a two-week acclimation period to minimize stress and ensure adaptation to the new environment.

Before colonization, the mice were treated with ampicillin (Sandoz) in their drinking water at a concentration of 0.5 mg/L for two weeks to facilitate the establishment of bacterial strains in the gut environment.

Colonization was then achieved via orogastric gavage. Each mouse received a dose of 10^8^ colony-forming units (CFUs) in 100 microliters of PBS for each bacterial strain. The bacterial strains used for colonization were MDR-KP, EC, EC-OXA, and EC-CTXM. The control group was treated with sterile PBS following the same protocol. Each experimental groups contained six mice. After orogastric colonization, ampicillin treatment through the drinking water (0.5 mg/L) was maintained until the end of the experiment. Fresh fecal samples were collected and weighed for further investigation on Days 5, 10, and 14 after colonization. For the determination of the germ count, samples were used immediately after collection. For ELISA and DNA extraction, separated samples were stored at −80°C until they were investigated.

During the acclimation period and throughout the experiment, mice were handled gently to maintain their welfare. Experimental procedures were conducted in compliance with ethical guidelines and approved by the institutional animal care and use committee. Animals were maintained and handled in accordance with the recommendations of the Guidelines for the Care and Use of Laboratory Animals and the experiments were approved by the Animal Care Committee of Semmelweis University (Permission No. PE/EA/60-8/2018, PE/EA/964-5/2018).

### 4.3. Determination of the Fecal Germ Count of Mice

Fecal shedding of the colonized bacterial strains was quantified by determination of germ count in feces. Freshly collected fecal samples were weighed and immediately suspended by mechanical dissection of fecal pellet with sterile inoculation loop in 1 mL sterile PBS (VWR, Hungary) followed by thorough vortexing to gain a homogenous suspension. The suspension was serially diluted tenfold, and twenty microliters from each dilution were streaked onto selective chromogenic agar plates and incubated overnight. The following day, colonies were identified by appropriate color and counted. The germ count was calculated based on the colony numbers and dilution factors, expressed as CFUs per gram of fecal mass. For the multiresistant *K. pneumoniae* (MDR-KP), Orientation CHROMagar plates containing 0.5 mg/L ciprofloxacin (Fresenius Kabi, Bad Homburg vor der Höhe, Germany) were used to selectively support their growth. For *E. coli* J53 (EC) and transconjugant *E. coli* strains (EC-OXA and EC-CTXM), *Enterobacteriaceae* CHROMagar plates containing 100 mg/L sodium-azide (Merck, Darmstadt, Germany) were used. Results were statistically compared with two-tailed Student’s *t*-test.

### 4.4. Determination of Total IgA and Defensin Levels in Stool by ELISA

Total IgA, murine beta-defensin 3, and murine alpha-defensin 5 were determined from mouse feces by commercial ELISA kits (MyBiosource, San Diego, CA, USA, MBS7725462, MBS7725303, and MBS7725358). Collected frozen fecal samples were thawed and weighed before they were suspended in PBS and vortexed thoroughly for 1 h at 4 °C. Suspensions were centrifuged at 2500 rpm for 10 min and the supernatants were used in further studies. Sandwich ELISA measurements were made according to manufacturer’s instructions. After stopping the reaction, optical density was measured at 450 nm and 690 nm as reference wavelengths. Results were calculated with a calibration curve gained from included standards. Lastly, total IgA and defensin content were calculated (mg/g or pg/g feces). Results were statistically compared using Wilcoxon rank-sum test.

### 4.5. Microbiome Composition with 16S Metagenomic Analysis

To investigate the effect of different colonizing bacterial strains on the composition of gastrointestinal microbiota of mice, stool samples were collected on day 14 after colonization. DNA was extracted from ~80 mg of feces using the ZymoBIOMICS DNA Miniprep Kit (Zymo Research, Irvine, CA, USA, D4300) according to manufacturer’s instructions. The V3–V4 region of the bacterial 16S rRNA genes were amplified by PCR. Dual indices (barcodes) and Illumina sequencing adapters were added to the amplicons using the Nextera XT Index kit (Illumina, Inc., San Diego, CA, USA), followed by DNA purification (Agencourt AMPure XP, Beckman Coulter, Brea, CA, USA). Individual barcoded DNA samples were then quantified with Qubit dsDNA HS Assay kit with Qubit 2.0 (Thermo Fisher Scientific, Waltham, MA, USA), quantified with DNA 7500 kit with Agilent 2100 Bioanalyzer (Santa Clara, CA, USA), normalized, and pooled. Multiplexed libraries were diluted to 7 pM and denatured with NaOH prior to sequencing on the MiSeq system (Illumina) using the MiSeq reagent kit v3 600 cycles (2 × 300 bp; Illumina). Results of the sequencing were uploaded and analyzed with the CosmosID-HUB software v2.0 [47]. Paired-end reads of samples were analyzed by DADA2 algorithm, after primer removal data were quality-trimmed with a threshold of median Phred score 20 over the length of reads. Forward and reversed reads were trimmed to a uniform length based on quality of reads and merged if they have at least 12 base long overlap followed by the removing of chimeric sequences. Data were then processed to amplicon sequence variants (ASVs). The taxonomical annotation of clusters was made using DADA2’s naive Bayesian classifier and the Silva version 138 database. Microbial composition of samples was characterized by relative abundance of identified taxa, diversity indices (CHAO1, Simpson), and comparison of abundance distribution of specified taxa between experimental groups. Statistical comparison was made using Wilcoxon rank-sum test.

## 5. Conclusions

In our study, a mouse model demonstrated that intestinal colonization with the MDR CTX-M-15- and OXA-162-producing *K. pneumoniae* ST15 high-risk clone is multifactorial. Not only the MDR clone itself but also the resistance plasmids, namely, IncFII(K) and IncL, play a primary role in the colonization rate in the gastrointestinal tract. The levels of IgA, beta-defensin-3, and alpha-defensin-5, as well as the intestinal microbiota composition influence the colonization of the MDR *K. pneumoniae* high-risk clone.

## Data Availability

The datasets supporting the conclusions of this article are included within the article. The sequencing data generated during the current study are available through following the figshare identifier: https://doi.org/10.6084/m9.figshare.26124370.
